# A status report on management of cleft lip and palate in India

**DOI:** 10.4103/0970-0358.63938

**Published:** 2010

**Authors:** A. Gopalakrishna, Karoon Agrawal

**Affiliations:** Department of Plastic Surgery, Deccan College of Medical Sciences and Allied Hospitals, Hyderabad, Andhra Pradesh, India; 1 Department of Plastic Surgery, JIPMER, Pondicherry, India

**Keywords:** Cleft lip and palate, cleft survey, cleft management protocol

## Abstract

**Introduction:**

This national survey on the management of cleft lip and palate (CLP) in India is the first of its kind.

**Objective:**

To collect basic data on the management of patients with CLP in India for further evaluation.

**Materials and Methods:**

A proforma was designed and sent to all the surgeons treating CLP in India. It was publicized through internet, emails, post and through personal communication.

**Subjects:**

293 cleft surgeons representing 112 centers responded to the questionnaire. Most of the forms were filled up by personal interview.

**Results:**

The cleft workload of the participating centers is between 10 and 2000 surgeries annually. These centers collectively perform 32,500–34,700 primary and secondary cleft surgeries every year. The responses were analyzed using Microsoft excel and 112 as the sample size. Most surgeons are repairing cleft lip between 3-6 months and cleft palate between 6 months to 1 year. Millard and Tennison repairs form the mainstay of lip repair. Multiple techniques are used for palate repair. Presurgical orthopedics, lip adhesion, nasendoscopy, speech therapy, video-fluoroscopy and orthognathic surgery were not always available and in some cases not availed of even when available.

**Conclusion:**

Management of CLP differs in India. Primary surgical practices are almost similar to other studies. There is a lack of interdisciplinary approach in majority of the centers, and hence, there is a need for better interaction amongst the specialists. A more comprehensive study with an improved questionnaire would be desirable.

## INTRODUCTION

The treatment of cleft lip and palate (CLP) spans a long period of growth in a child and the outcome of treatment is influenced by multiple factors. It is desirable to provide a few set protocols for service providers and to have a nationwide evaluation of the outcome of such protocols over short-term and long-term.

On reviewing the literature from India, it was found that the publications tended to be about technical details or of a general nature about CLP.[1‐3] Many studies have been conducted in Singapore,[4] UK,[5‐10] USA,[11‐13] Korea,[14] Brazil,[15] Thailand,[16] etc. These surveys dealt with the organization of services for cleft lip and palate, availability of care, effects of previous surveys and importance of specific management techniques in the respective countries.

To generate the basic data on existing facilities, management protocols and treatment modalities for CLP in India a national survey of service providers was carried out. It does not focus on the outcome of the treatment.

## MATERIALS AND METHODS

This survey was conducted from May 2006 to September 2007. Approximately 970 letters and questionnaires were sent to the members of Indian Society of Cleft Lip, Palate and Craniofacial anomalies and Association of Plastic Surgeons of India. The authors used the portal "plastic_surgery@yahoogroups.com" of India and publicized this survey many times at periodic intervals and also during various opportunities at workshops, seminars and national and international conferences held in India. During these events, personal meetings were organized with the respondents and they were motivated to fill up the survey forms. During the survey, the ethical principles for medical research involving human subject as outlined in the world medical association declaration of Helsinki were followed.

This survey was performed through a standard questionnaire. The questions were simple, easy to understand and concise enough for the respondents. In majority of the questions, the possible options were given for ease of selection. There were 28 questions which required 6–7 minutes on trial. There was a confidential introductory page for the details of the respondents which was optional. Some of the questionnaires were filled out on behalf of the institutes or the departments, where more than one surgeon were working. A total of 112 filled up survey forms could be collected with the responses of 293 surgeons.

Some questions have been answered by ticking multiple alternatives by some respondents indicating either different choices of different surgeons in the same center or that an individual may vary his approach from time to time. Statistical analysis was performed using preloaded Microsoft Excel standard software. The data was fed in the master chart and the percentage was analyzed. The number of responses to various questions was variable. Hence, the number of the responses received (n = 112) has been used as the denominator for the analysis.

## RESULTS

**Figure 1:**
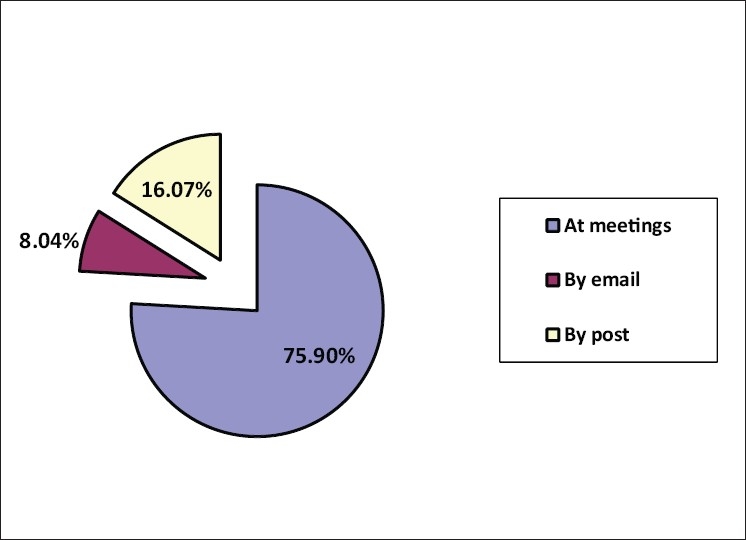
A pie diagram showing the modality of receipt of the responses in the survey

**Figure 2:**
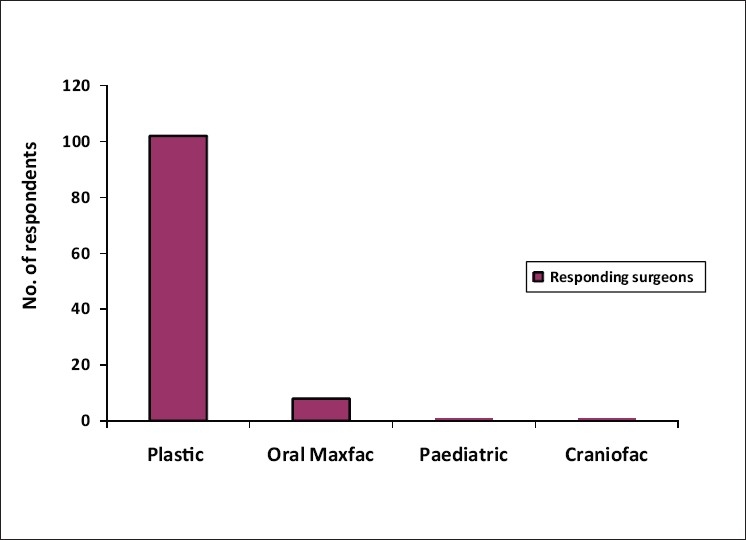
A bar chart on the specialty of the respondents of the survey

**Figure 3:**
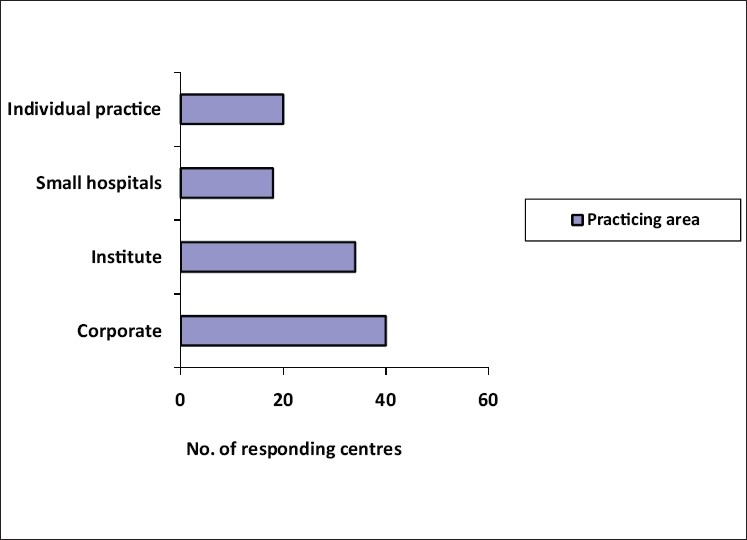
Pattern of practice of participating surgeons in the survey

**Figure 4:**
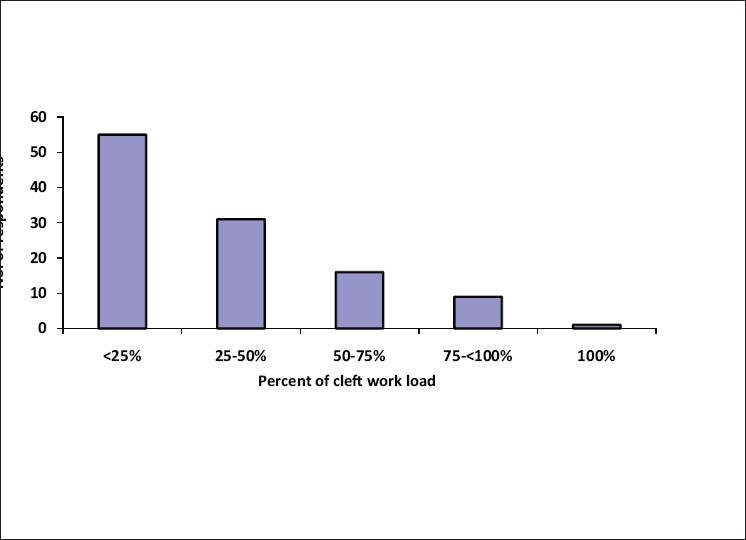
Cleft work load of responding centres in the survey

The response to the questionnaires by post and email was lukewarm. 85 (75.9%) forms were filled up by the respondents during one to one meeting on the sidelines of scientific meetings over a period of 16 months [Figure 1]. Of the 112 questionnaires received back, 102 were from plastic surgeons, a few from maxillofacial surgeons and a pediatric surgeon [Figure 2]. The responding cleft surgeons are working in 34 medical colleges, majority of which are government institutions and the rest working in corporate and trust hospitals, smaller hospitals and private clinics [Figure 3]. The number of patients operated upon by the responding centers has been enlisted in Table 1. Notably, there are five centers performing 1,000-1,500 cleft surgeries annually. The respondents collectively claim to perform approximately 32,500–34,700 surgical procedures for cleft lip and palate annually. This includes primary lip and palate repair as well as the secondary procedures such as palate fistula closure, surgical management of velopharyngeal incompetence, orthognathic surgery, rhinoplasty and revision procedures. Cleft work load of respondents has been given in Figure 4.

**Table 1:** Number of cleft surgeries performed by the participating cleft centres per annum

*Number of patients*	*Number of respondents*
No mention of the number of patients	8
<20	5
21-50	16
51-100	11
101-200	19
201-300	10
301-400	9
401-500	15
501-1,000	14
>1,000	5
Total	112

**Figure 5:**
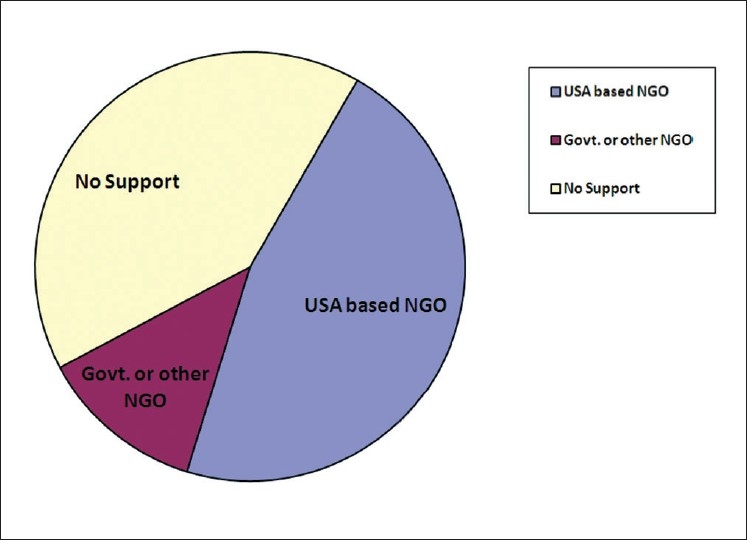
A pie diagram showing the type of support to the participating organizations in the survey

The respondents received financial support from a US-based non-government organization (NGO) exclusively sponsoring cleft lip and palate surgeries, their own institution or from other non-government organizations [Figure 5]. Majority of the state and central government sponsored Institutions did not receive financial support from NGOs.

### Feeding

Spoon feeding is the most popular method of feeding the cleft lip and palate children in India. 101 (90.2%) Indian surgeons advise using the spoon as the primary feeding appliance. There are 22 (19.64%) who advise feeding appliances such as shaped containers, droppers, etc. In different parts of India, many indigenous types of feeding appliances are used even for normal children which are modifications of a spoon. The authors believe that though some of the surgeons stated spoon feeding, however, some parents may have been using these indigenous appliances. Rarely, the surgeons advise a feeding bottle. Two of them, interestingly, insist on breast feeding.

### Presurgical orthopedics

A total of 19 (16.96%) respondents applied presurgical orthopedics in cleft care. They use NAM 5 (4.46%), Latham a 5 (4.46%), both 4 (3.57%), 4 (3.57%) use other techniques and one (0.89) did not specify. 82 respondents (73.21%) do not subject their patients to presurgical orthopedics. Amongst these respondents 57 (69.51%) do not do so because facility and manpower for fabricating the appliances are not available and 15 (18.29%) do not prefer presurgical orthopeadics. 10 (12.2%) did not specify.

### Prerequisite for surgery

There seems to be a reasonable agreement on the minimum hemoglobin of 10 gms%, with only 14 (12.5%) centers willing to operate between 8 and <10 gms%. Criteria of minimum weight of the child for cleft repair were highly variable. The responses have been listed in Table 2. Majority wanted the child to weigh at least 4-5 kg before surgery. Preoperative throat swab culture and sensitivity does not seem to be very popular. Interestingly, there are different verdicts in cleft lip and palate. In lip surgery, only 8 centers (7.14%) perform throat swab culture, however, in cleft palate 33 (29.44%) respondents examine the throat swab for culture and sensitivity.

**Table 2:** Minimum preoperative body weight of the child with CLP

*Minimum body weight (in Kg)*	*No. of the responding centre*	*Percentage*
No answer	46	41.07
3	2	1.79
4	8	7.14
4.5	20	17.86
5	28	25
5.5	1	0.89
6	2	1.79
7	2	1.79
7.5	1	0.89
8	2	1.79
Total	112	100

### Timing of repair

Majority of the surgeons prefer to repair lip before 6 months and preferred age for cleft palate repair is 6-12 months [Table 3].

**Table 3:** Timing of unilateral and bilateral cleft lip repair and palatoplasty by the respondents

*Surgery*	*≤3 months*	*>3-6 months*	*>6-12 months*	*>12-18 months*	*No answer*
Cleft lip					
Unilateral (n = 112)	17 (15.18)	92 (82.14)	3 (2.68)	0	0
Bilateral (n = 112)	12 (10.71)	73 (65.18)	12 (10.71)	2 (1.79)	13 (11.6)
Cleft Palate	0	0	85 (75.89)	26 (23.21)	1 (0.89)

Figures in parentheses are in percentage

### Lip adhesion

82 of the respondents (73.21%) do not advocate lip adhesion as a preliminary procedure. However, it is used by 5 (4.46%) respondents for wide unilateral cleft lip and 10 (8.93%) would use it for a bilateral cleft lip. The questionnaire did not specify about protruding premaxilla.

### Techniques of cleft lip repair

Millard's Rotation Advancement technique with various modifications is the most popular technique for lip repair. 58.1% and 44.7% responding surgeons prefer this technique for unilateral and bilateral cleft lip repair respectively. There is a general agreement on repairing both sides of the bilateral cleft lip simultaneously [Table 4].

**Table 4:** Preferred techniques of unilateral and bilateral cleft lip repair in various studies in literature

**	*Korean study[14]*	*Weinfield et al., 2005[17]*	*Eurocleft 2005[18]*	*Sitzman et al., 2008[13]*	*Present study*
**	*Percent*	*Percent*	*Centers (n = 5)*	*Percent*	*Percent*
Unilateral					
Rotation	100	84.2	2 centers	84 (45% modify)	58.1
Advancement					
Triangular flap			2 centers	9 (Variant)	39.5
and		15.7#			
Straight line					
Skoog			2 centers		
Unspecified			1 center		2.4
Bilateral					
Simultaneous	90				93.8
Rotation	65	72			44.7
Advancement					
Mulliken	21				
Noordoff	15*				
Veau	5*				
Black	2*				10.5
Straight line					35.09
Triangular		28#			
Thompson					2.1

* As per the bar diagram in the article

# combined data for straight line repair and triangular flap technique

### Primary nasal correction

In unilateral cleft lip primary nasal correction at the time of lip repair is commonly done. 91 (81.25%) respondents favor primary ala correction. Only 45 (40.17%) are performing primary nasal correction in bilateral cleft lip.

### Technique of cleft palate repair

Indian surgeons seem to utilize all the techniques of palatoplasty equally [Table 5]. 69 (61.61%) of the respondents do not fracture the pterygoid hamulus routinely. In response to the question on management of secondary raw area in the hard palate, 46 (41.07%) of the respondents pack the lateral secondary raw area in the palate. It is interesting to note that 5 (4.46%) respondents do not leave any raw area. One of them did not respond to this question.

**Table 5:** Various techniques of cleft palate repair preferred in different studies in literature

	*Korean study, 2003[14]*	*Weinfield et al., 2005[17]*	*Eurocleft, 2005[18]*	*Present study*
**	*Percent*	*Percent*	*Center*	*Percent*
One stage repair of soft + hard palate	95	97.1		94.17
Pushback	64	30.3	*	41.07
Furlow	43	34.6		6.43
Two Flap		14.4		41.97
IVV		20.4		30.36
Langenbeck			*	
Kriens			*	
Others				6.43

To a specific question on anchorage of palatal mucoperiosteal flaps, surgeons from 56 (50%) centers anchor the palatal flaps to the nasal mucosa, 10 (8.93%) anchor them to the alveolar margins and 5 (4.46%) attach them to the palatal shelves only. However, surgeons of 20 (17.86%) centers prefer to anchor the flap to the nasal lining and the alveolar margin, 4 (3.57%) to the nasal lining and the palatal shelves, while one (0.89%) surgeon anchors to all the three possible attachments and 10 (8.93%) respondents do not anchor the flaps at all.

### Simultaneous cleft lip and palate repair

62 respondents (55.36%) are willing to perform one stage repair of cleft lip and palate, whereas the rest are not. Those who repair in one stage are almost equally distributed over the timing of repair at 1 year - 1 1/2 years (16.07%), 2 years - 23 (20.54%), and older - 21(18.75%). A specific question was asked whether the surgeon was willing to offer one stage repair to a patient seen soon after birth. Nearly half of them i.e., 30 (26.79%) respondents would like to offer one stage repair at first counseling.

### Secondary surgery

#### Fistula repair

For fistula repair local flaps were the overwhelming favorites with 98 (87.5%) respondents. 45 (40.18%) use tongue flap, 22(19.64%) respondents use buccal flap and two surgeons preferred redoing the palatoplasty.

#### Pharyngoplasty

On being asked for percentage of patients requiring pharyngoplasty 26 (23.21%) said 0-10% of their cases require pharyngoplasty. 16 (14.3%) said that 10-20% require pharyngoplasty. 8 (7.1%) felt that 20-30% of their patients required pharyngoplasty. One respondent felt the need for pharyngoplasty in over 50% cases. The question appeared to be unpopular as less than half of the surgeons responded to this question.

The question on 'preferred technique of pharyngoplasty' evoked a good response. Superiorly based pharyngeal flap pharyngoplasty is the most popular technique for correction of the velopharyngeal incompetence [Table 6].

**Table 6:** Pharyngoplasty procedures used by the respondents in the various studies for the management of velopharyngeal incompetence

	*Korean study, 2003[14]*	*Weinfield et al., 2005[17]*	*Present study*
Superiorly based pharyngeal flap (%)	71	34.5	50
Hyne's/Sphincter (%)	-	-	8.93
Inferiorly-based pharyngeal flap (%)	-	-	8.04
Palatoplasty (%)	-	65.5	
Unspecified (%)	-	-	7.14

#### Orthognathic surgery

The response to the question on availability of orthognathic surgery was lukewarm, as only 69 (61.6%) responses were received. However, 29 (25.8%) do not perform osteotomies. Rest of the respondents of this question felt that less than 10% of their patients required maxillary osteotomy. The other surgeons probably do not have access to orthognathic surgery.

#### Availability of facilities

93 (83.04%) cleft surgeons have access to speech therapy. Availability of orthodontic support, videofluoroscopy and nasendoscopy has been given in Table 7. However, 84 (75%) responses were available to the question on the percentage of patients undergoing speech therapy. Detailed break-up of the patients requiring speech therapy is poedrtrayed in Table 8.

**Table 7:** Availability of rehabilitation facilities for cleft palate patients in Indian centres during the follow-up period

*Facility*	*Available (n = 112)*	*Percentage*
Speech therapy	93	83.04
Orthodontia	84	75
Nasendoscopy	48	42.86
Video-fluoroscopy	37	33.04

A total of 48 (42.86%) respondents perform nasendoscopy. 24 out of these 48 do it at the age of 4-8yrs; only two respondents perform it in patients under 4 years of age, while one respondent does it for patients older than 10yrs. Rest of the respondents did not answer the age factor.

## DISCUSSION

This type of survey has been conducted in India for the first time. It is a huge survey considering that 293 surgeons working in 112 centers have participated in this study. An attempt was made to avoid any bias in the selection of the respondents. All the members of the Association of Plastic Surgeons of India and the members of Indian Society of Cleft Lip, Palate and Craniofacial Anomalies were invited to participate. This covers a majority of the cleft surgeons in India. To disseminatae the information further, yahoo group was used for announcements and reminders. Initially the response to the postal and email request was poor. In an attempt to get a better response, the authors collected the proformas during personal meetings. More responses were received from the relatively high volume cleft surgeons and surgeons attending conferences and meetings. Being plastic surgeons, the authors had better contacts with plastic surgeons. Also, majority of the cleft surgeons in India are plastic surgeons. A few maxillofacial surgeons and pediatric surgeons perform cleft surgeries. Technically there may be a bias in selection of the respondents, however, this was unintentional.

In UK, the health ministry constituted clinical standards advisory group (CSAG) to investigate the standard of care of cleft lip and palate in UK, the health needs of cleft lip and palate children and to compare the quality of care provided by high and low volume providers and suggest changes in the light of their findings.[17] This being a preliminary study, the authors decided in favor of assessing only the infrastructure and the protocol of cleft care in different centers in India.

An international survey was conducted in 2004 by sending questionnaires to 224 cleft centers in USA and abroad. Collectively, the responding surgeons claimed to be managing 6,432 new patients annually.[19] In the present survey, our 112 participating centers claim to perform approximately 32,000-34,700 surgeries per annum, including primary repairs and secondary procedures. This number is unmatched by any other publication in the literature, moreover this does not cover all the surgeries being done nationwide. There are very interesting observations in some of the areas in this survey, which are worth discussing.

Spoon feeding is preferred over bottle feeding by a majority of surgeons and some use feeding appliances other than the bottle. In contrast, in a randomized controlled trial organized in a Brazilian hospital, feeding bottle was used in an overwhelming majority (92%) of infants and feeding cup, an spoon, dropper and syringe were used in very few. For 72% of babies feeding tube was used for sometime.[20] A postal survey conducted by Oliver and Jones (1997) indicated preponderance of bottle feeding in 64-90% in different types of cleft lip and palate children.[21] The feeding practices of children with CLP seem to be entirely different in India with respect to the other countries. This is because of the basic teaching in our country. Bottle feeding is not encouraged by the pediatric physician as it requires cleaning and asepsis. Indian parents living in slums and rural areas may not be able to maintain a very good hygiene with a bottle. New feeding appliances are expensive and not easily available in India. Hence feeding with spoon and indigenous appliances is preferred over bottle feeding.

Presurgical orthopedic appliances are not very popular. Naso-alveolar moulding (NAM), Latham and other techniques are in use equally amongst the respondents. Most of the cleft centers do not have the facility and manpower to do so. Majority of the surgeons would like to use the presurgical orthopedic appliances if available within the financial reach of the parents. There is a significantly high response in favor of presurgical orthopedics in other studies.[14,17] There are very few orthodontists available in India dedicated to cleft care. Also, most of Indian cleft surgeons lack training and exposure to orthodontic appliances. Because of these factors preoperative orthopedics is yet to catch up in India and remains an area of major concern in cleft care.

Majority of the respondents agree with the universal preoperative requirement of at least 10 gm% hemoglobin and minimum weight of 4-5 kgs in CLP. But few respondents are willing to operate on up to 8 gm% hemoglobin. The Indian cleft surgeons receive a large number of anemic and underweight children. In these children waiting for improvement is likely to delay the surgery or they are likely to drop out from the clinic. Therefore in some of the centers children are accepted for surgery with lesser hemoglobin and lesser body weight. Guruwardana et al. (1999) have shown no significant difference in perioperative morbidity between healthy children with hemoglobin values 7-10 gm% and those above 10 gm%.[22] Anemia, underweight, poor socioeconomic status, limited monitoring facilities, shortage of resources for anesthetic drugs etc call for innovative skill on the part of the anesthetists for delivering safe anesthesia to these patients.[23]

As per the present survey lip adhesion is not a popular procedure (4.46% in UCL and 8.93% in BCL). However, lip adhesion is used in a majority of unilateral as well as bilateral cleft lip.[14,17] This procedure increases the number of surgeries in the management of cleft lip which may not be favorable in the Indian scenario. Most of the Indian surgeons probably the that lip adhesion does not improve the overall result of lip repair.

There is a broad agreement on the timings of unilateral cleft lip repair amongst the participating centers [Table 3]. In all the studies there is a consensus regarding the age of unilateral cleft lip repair before 6 months. But this is not true for bilateral CL. There are 12.5% survey responses preferring relatively late repair of BCL after 6 months. The trend for relatively late repair is probably because of the relatively poor nutritional status and low body weight of these children, more so in bilateral cleft. Majority (75.89%) of the centers in the present survey prefer to perform palatoplasty between 6-12 months, however, almost all the centers intend to complete palatoplasty by 18 months of age.

As per the present survey Millard's rotation advancement and triangular flap repair form the mainstay of surgical correction of unilateral cleft lip, Millard's repair and straight line repair form the bulk of procedures for bilateral cleft lip. In personal communications, most respondents have mentioned that they do make many variations in the classical surgical procedures as described. "We learn the technique from our teachers, who incorporate their own slight modifications, and then over the years modify it further ourselves", was how one of the respondents put it.

There is a reasonable variation in the preferred technique of unilateral cleft lip repair amongst various studies [Table 7]. The triangular flap has a good following in India (39.5%) because this technique has very deep root in our country. Almost all previous generation teachers were trained in triangular flap technique and its variations. New generation surgeons are being trained abroad and have started practicing rotation advancement technique more often. Still the Indian surgeons tend to incorporate a triangle somewhere in the course of repair with the intention to break the straight suture line from the columella base to the vermilion. Primary nasal correction at the time of unilateral cleft lip repair is very commonly (81.25%) done in India. This is at par with Weinfield et al. international survey (2005) in which 88.3% surgeons perform primary alar repositioning.[17]

Most of the cleft surgeons use different techniques of palatoplasty depending upon the type of the cleft palate. However, the question in the survey was not framed for different types of the cleft palate. Hence, the data is a collective data for all types of clefts. In future, we need to ask the preferred technique of palatoplasty for each type of cleft palate. Furlow's double opposing Z-plasty is not a popular technique (6.43%) for CP repair in the present survey while in the Korean study[14] (43%) and in Weinfield et al. international survey[17] (34.8%) it was a popular technique. In the present survey pushback, two-flap technique and intervelar veloplasty were nearly equally preferred [Table 8]. There could be a little confusion in the use of nomenclature of these techniques. Fracture of the pterygoid hamulus seems to have been given up by more than half of the Indian surgeons (58.93%). To a specific question regarding the management of lateral secondary raw area in the palate, a large number of Indian surgeons still use the pack. It is interesting to know that 5.98% respondents do not leave a raw area after palatoplasty.

**Table 8:** Percentage of patients subjected to speech therapy in children with cleft palate in Indian cleft centers

*Requirement of speech therapy*	*No. of respondents (n = 112)*	*Percentage*
No answer	28	25
<10	12	10.71
10-20	8	7.14
>20-40	11	9.82
>40-60	14	12.5
>60-80	6	5.36
>80-100	33	29.46

One stage repair of cleft lip and palate has many takers (55.36%) in our survey. There are a number of centers willing to offer one stage repair to a patient if seen soon after birth. These centers will prefer to perform a one stage surgery between one to two years or even later. This concept of one stage cleft lip and palate repair is unique to India. This was initially designated as 'hole in one' by Dr. C. J. T. Pinto, an avid golfer and the Indian plastic surgeon who introduced it. Now-a-days it is popularly known as 'Whole in one', a term more easily understood. Late arrival of cleft patients and drop out after cleft lip repair is quite common. There are a large number of patients from the low socioeconomic status. There is a usual tendency to avoid treatment of children with deformities in rural India. Hence, the drop out is extremely common. Some of the surgeons started performing combined one stage palatoplasty and lip repair in the late arrivals.[24] Lately, some of the surgeons have started using this technique of one stage in children of one year or above. This seems to be working well in some centers. This definitely reduces the financial and time burden on the family. However, this delays the first surgery, is difficult to use in relatively underweight and anemic children and two simple surgeries combined together become a major intervention with long anesthesia time. At present this is matter of debate in India during most of the scientific meetings. This option has not been well-documented in the literature.

Palatal fistula does not seem to be a part of earlier surveys in the literature. In the present survey, local flaps and tongue flaps were the mainstays of the management of this complication. One surgeon preferred to redo the palatoplasty for the management of majority of palatal fistula.

The question on "percentage of patients requiring pharyngoplasty" did not seem to be popular. Very few responses were received. The preferred technique of pharyngoplasty was superiorly based pharyngeal flap pharyngoplasty (64%) as in the Korean study[14] (71%) [Table 4].

Only 17 responses were received regarding the need for maxillary osteotomy in the present survey. This is possibly because most respondents were plastic surgeons and in India, traditionally the majority of the Indian plastic surgeons are not trained for nor do they perform orthognathic surgeries. Another reason for poor response may be the poor follow up of these patients in majority of Indian cleft care centers. With the involvement of oral and maxillofacial surgeons in the recent past, some of the centers have started performing cleft orthognathic surgeries.

In the present study, 81.8% of the respondent surgeons had access to speech therapy. But to the question of percentage of patients requiring speech therapy, the data may indicate the compliance/non-compliance of patients to the advice of speech therapy. On personal discussion with the respondents, the surgeons who responded in favor of 80-100% patients undergoing speech therapy stated that the speech therapist was a part of the team within the institute and almost all the cleft patients were attached to speech therapist as a routine. Hence, the response in the present study does not indicate the result of palate repair in terms of speech result after palatoplasty. In Korea, 98% acknowledged referral for speech pathology with 40% referring "always" and 52% "frequently" and 14% of the programs had team approach in the management.[14]

Nasendoscopy is becoming popular in India as almost 39.09% of respondents have the facility. It is significantly less than in the international[11] and USA surveys.[17] Surprisingly, the video fluoroscopy also seems to be reasonably popular in India as a large number of respondents have this facility [Table 9].

**Table 9:** Percentage of nasendoscopy and video-fluoroscopy performed for evaluation of VPI by respondents of various surveys

*(%)*	*USA survey, 1993 (n = 27)[11]*	*International survey, 2005[17]*	*Present study (n = 112)*
Nasendoscopy	90	79.45	39.09
Video-fluoroscopy		20.6	30.91

In a study of this kind, there are certain inherent weaknesses. For example, what the questioner is seeking may not always be crystal clear from the question as it is framed. However, this was overcome by one-to-one interaction with the respondents. The second problem is that the respondents tend to give guarded answers to potentially controversial issues, as observed by low responses to some of the questions, in spite of assurance of anonymity. Another problem is that answers were given without reference to statistics of their respective departments. The authors present the responses received at their face value because they do not have a mechanism for authenticating the responses. However, in a large sample of this kind they hope that minor discrepancies will not be statistically important. The highlights of this survey have been showed in Table 10.

**Table 10:** Highlights of the Indian survey

1.	Feeding practices of CLP children is different in India. Spoon feeding is the norm.
2.	8-10 gms hemoglobin is acceptable for surgery.
3.	Presurgical orthopedics and orthognathic surgery are not yet popular.
4.	Triangular flap is almost as popular as Millard's technique for cleft lip repair.
5.	Bilateral cleft lip is repaired relatively later than the unilateral cleft.
6.	One stage repair of cleft lip and palate is gaining popularity.
7.	Push back and two flap palatoplasty are preferred techniques.
8.	Furlow palatoplasty is not a popular technique.
9.	Superiorly based pharyngeal flap pharyngoplasty is a technique of choice for the management of VPI.
10.	Speech therapy and Orthodontic care are commonly not available.

In a first of its kind survey, the authors have analyzed the management of cleft lip and palate in India. Improvement in economy, education, transport, and health infrastructure are resulting into better cleft care. Involvement of non-governmental organizations is very rapidly changing the cleft care scenario in the country irrespective of the economic status. The authors hope that this survey will provide an impetus for further inter-center, zonal, regional, and national surveys on various aspects of cleft management in India, so as to improve the overall care of cleft patients and bring them to main stream society.
